# Partial hepatectomy for treatment of multiple liver abscess in a calf: a case report

**DOI:** 10.1186/s12917-021-02750-y

**Published:** 2021-02-03

**Authors:** Reiichiro Sato, Kazutaka Yamada, Taiki Yokoyama, Koki Tanimoto, Shoko Takeuchi, Natsumi Tatsuzawa, Shiho Nakui, Hiroyuki Satoh, Mahmoud Fadul, Adrian Steiner

**Affiliations:** 1 Faculty of Agriculture, University of Miyazaki, 1-1 Gakuen Kibanadai-nishi, Miyazaki, 889-2192 Japan; 2 Faculty of Veterinary Medicine, Azabu University, 1-17-71 Fuchinobe, Chuo-ku, Sagamihara, Kanagawa 252-5201 Japan; 3 Clinic for Ruminants, Vetsuisse-Faculty, University of Bern, Bremgartenstrasse 109a, 3012 Bern, Switzerland

**Keywords:** Calf, Computed tomography, Ligasure, Liver abscess, Omphalophlebitis, Partial hepatectomy, Ultrasonography

## Abstract

**Background:**

Umbilical vein bacterial infections may cause liver abscesses during bacterial ascent. A single liver abscess can be surgically treated by marsupialization, but a risk of recurrence or non-healing remains. Moreover, there is no effective treatment for multiple abscesses.

**Case presentation:**

A 17-day-old Holstein female calf exhibited reduced general condition, swelling and drainage of the umbilicus, and pressure sores in the area of the carpus, resulting in reluctance to stand up. The umbilicus showed pain at palpation; deep abdominal palpation indicated a swollen umbilical vein coursing from the umbilicus toward the liver. Ultrasonography confirmed a swollen umbilical vein with pus accumulation and multiple abscesses in the liver. Contrast-enhanced computed tomography (CT) examination confirmed that the swollen umbilical vein with fluid continued to the liver, and multiple unenhanced lesions, most likely abscesses, were confirmed in the liver. Partial hepatectomy was performed to remove as many abscesses as possible. For the resection, a vessel sealing device (LigaSureTM) was used to excise a part of the left liver lobe. As we could not remove all the abscesses in the liver during the operation, cefazolin sodium (5 mg/kg) was administered for 14 days after surgery. Post-operatively, blood accumulation was observed in the abdominal cavity, but no signs of peritonitis were found. The calf returned to the farm on day 38 after surgery. Follow-up information was obtained after 1 year, and complications were not reported.

**Conclusions:**

To our knowledge, this is the first report of partial hepatectomy using a vessel sealing device for a calf with multiple liver abscesses. This case report suggests that the combination of partial hepatectomy and long-term administration of antibacterial drugs may restore the health of calves with multiple liver abscesses.

## Background

The umbilicus comprises one umbilical vein, two umbilical arteries, and one urachus [1, 2] and plays important roles in normal fetal development for the delivery of oxygen-rich blood and nutrients from the dam to the fetus, the discharge of waste products, and transport of oxygen-poor blood from the fetus back to the dam [1, 3, 4].

The umbilical cord regresses spontaneously after birth but may persist when it is disrupted improperly in the course of dystocia or cesarean section. Moreover, it may get infected in cases of an unhygienic delivery environment, insufficient disinfection of the umbilicus, or deficiency of passive transfer of immunoglobulins [5–8].

In the umbilical cord, infection of the umbilical vein is second only to that of the allantoic tube (urachus) and is said to occur in 1–14% of newborns [9]. The umbilical vein enters the liver from between the left lobe of the liver and the quadratic lobe, and then joins the portal circulation; thus, bacteria infecting the umbilical vein may ascend to the liver to form a liver abscess. A calf with a liver abscess presents with fever, reduced general condition, and poor growth [2, 4]. Finally, when bacteria are disseminated systemically through the blood circulation, arthritis and pneumonia may occur, resulting in a poor prognosis or death. Omphalophlebitis is, thus, a disease that causes considerable losses [10, 11].

Although there are reports for single liver abscess by marsupialization [5, 6] and partial hepatectomy [12], there is no effective treatment for multiple liver abscesses. Here, we report a case of multiple liver abscesses in a calf treated with partial hepatic resection.

## Case presentation

A 17-day-old Holstein female calf was presented with reduced appetite and bad general condition, swelling and drainage of the umbilicus, and pressure sores of the skin in the carpal area because of difficulty in standing up smoothly over a long period of time. The heart rate, respiratory rate, and rectal temperature were 130 beats/min, 60 breaths/min, and 38.5°C, respectively. The umbilical swelling was 4.0 x 4.0 x 4.5 cm in size and showed pain at palpation; deep abdominal palpation revealed a swollen umbilical vein coursing from the umbilicus to the liver.

The complete blood count was in the normal range (white blood cell count, 10,700 cells/μL; red blood cell count, 596 x 10^4^ cells/μL; and thrombocytes, 66.8 x 10^4^ cells/μL). The serum fibrinogen concentration of 900 mg/dL (reference range, 200–700 mg/dL) was high and the total protein concentration of 3.9 g/dL (reference range, 6.74–7.49 g/dL) was low.

**Fig. 1:**
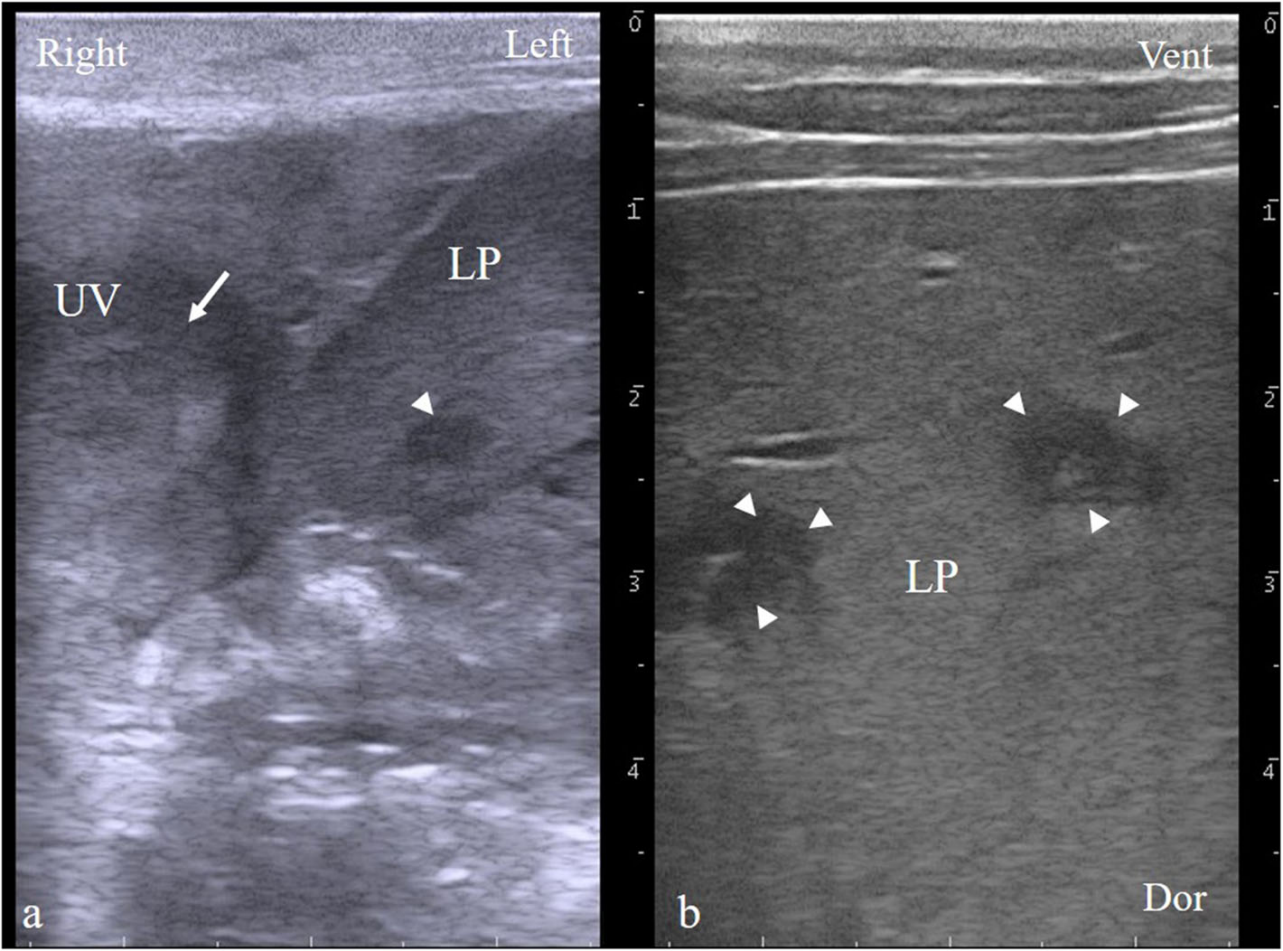
Ultrasound imaging (transverse plane) with a 10.0-MHz liner probe demonstrating the infected umbilical vein. **a** Scanner placement cranial from the abdominal fundus. The umbilical vein showed a horizontal diameter of 25 mm and a vertical diameter of 30 mm. The lumen of the vein had an area of relatively high echogenicity (arrow). **b** Scanner placement at the right 11^th^ intercostal space showing a hepatic abscess larger than 1 cm (arrowhead). LP, liver parenchyma; UV, umbilical vein; Vent, ventral; Dor, dorsal

Ultrasonography (13.0-MHz linear probe, MyLab One VET, Esaote, Maastricht, The Netherlands) confirmed the swollen umbilical vein (2.0 x 1.0 cm in size) with pus accumulation and revealed multiple abscesses dispersed in the liver (Fig. 1). The umbilical artery and the allantoic canal were only visualized as a thin duct.

Under sedation with intravenous xylazine hydrochloride (0.01 mg/kg), the calf was subjected to contrast-enhanced computed tomography (CT) scanning using a helical 80-row multi-detector CT device (AquilionPrimeSP/SPREAD, Canon, Ohtawara, Japan) for preoperative evaluation of the positional relationship of the umbilical vein to the liver and the liver abscess. Iopamidol (370 mgI/mL; Iopamiron Inj., Bayer, Leverkusen, Germany) was used as a contrast agent. Similar to the ultrasonographic findings, the CT images depicted the presence of the umbilical vein containing fluid coursing to the liver.

**Fig. 2:**
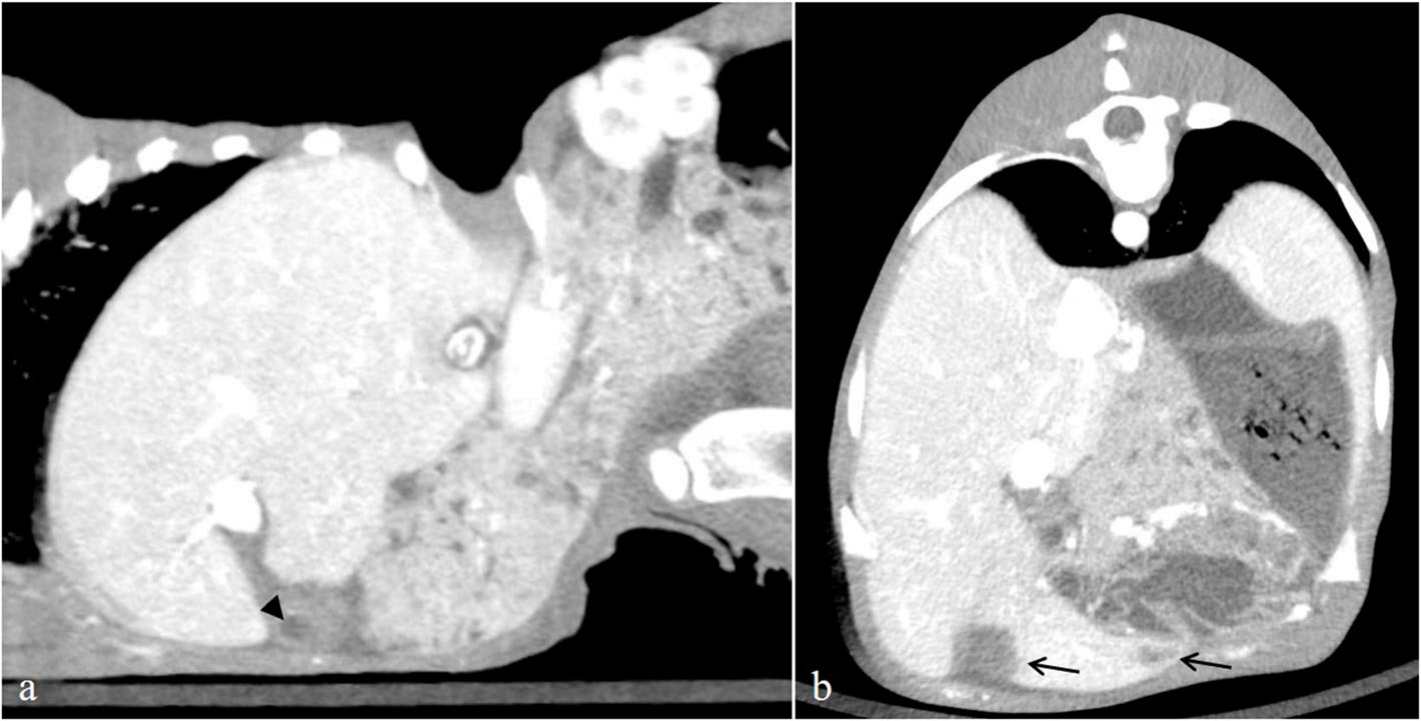
Contrast-enhanced CT images in the liver in the calf. **a** Sagittal image revealed an umbilical vein containing fluid (arrowhead). **b** Transverse image revealed multiple unenhanced lesions, most likely abscesses, which were confirmed in the liver (black arrow). CT, computed tomography

Moreover, multiple unenhanced lesions, most likely abscesses, were confirmed in the liver (Fig. 2). Furthermore, CT showed the size of the abscess, its position in the liver, and its positional relationship with the main blood vessels, such as the portal vein, hepatic artery, and hepatic vein in the liver parenchyma. In addition, CT is more useful than ultrasound because the liver can be stereoscopically imaged. In addition, the carpal joint had no evidence of inflammation; mild swelling of the soft tissues around the joint and a skin defect were confirmed.

T The referral veterinarian provided antibiotics for 10 days, but there was no improvement in the calf’s general or local condition, which worsened because the abscess was growing. Therefore, medical treatment was necessary. As there were multiple abscesses in the left lobe of the liver and two of them were close to the edge of the liver and separated from each other, it was considered difficult to be cured with marsupialization. Partial hepatectomy performance was decided to remove as many abscesses as possible from the liver.

The calf was feeding in the pen and had free access to water at all times. Nevertheless, the calf was fasted for 12 h prior to the surgery, and cefazolin sodium (5 mg/kg) (Cefazolin-Chu; Fujita Pharmaceutical, Tokyo, Japan) was administered intravenously to prevent perioperative infection. Then, the calf was positioned in dorsal recumbency and anesthetized by continuous administration of isoflurane (Isoflu; Zoetis Japan, Tokyo, Japan) at a concentration of 2% in oxygen. Local anesthesia with procaine hydrochloride (Kyoritsu Seiyaku Corporation, Tokyo, Japan) was administered subcutaneously around the umbilical and the right paramedian area. Preoperatively, the umbilical opening was closed with purse-string sutures and covered with a sterile glove.

**Fig. 3:**
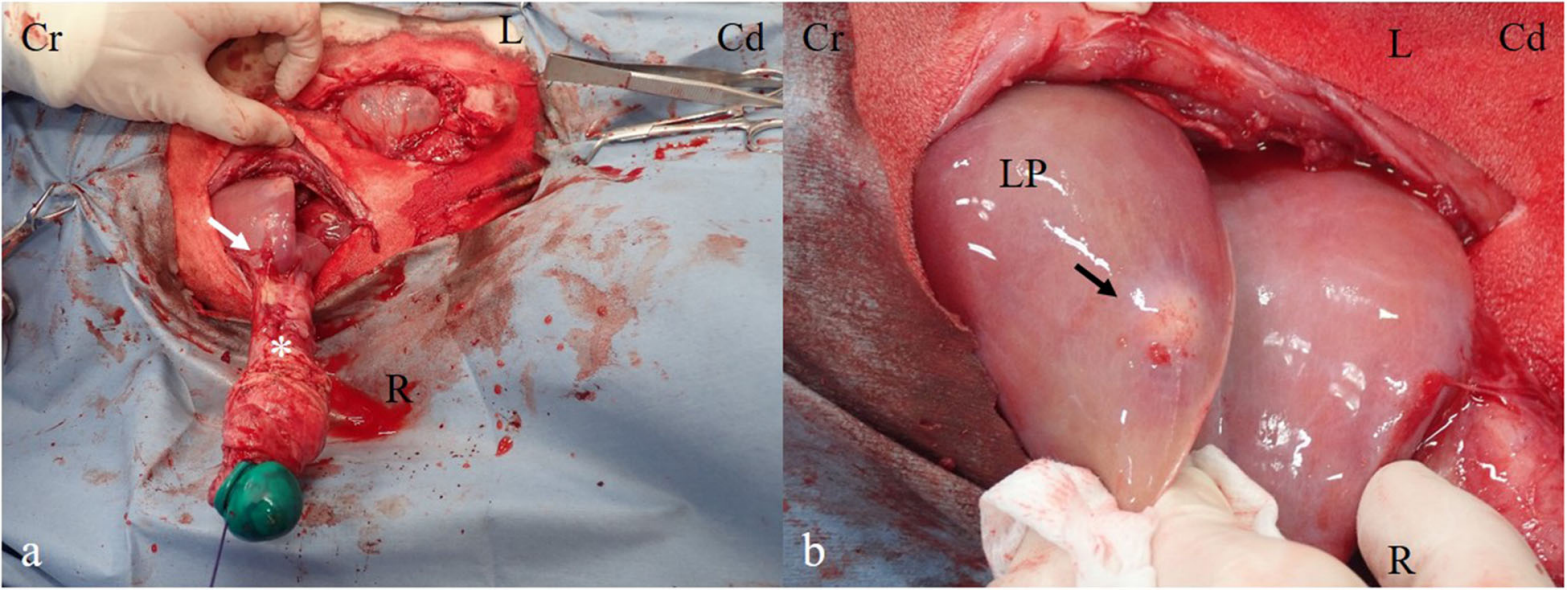
Intraoperative views of a partial hepatectomy. **a** Intraoperative appearance of the infected umbilical vein (*) penetrating the liver (white arrow). **b** Intraoperative appearance of the abscess surrounded liver parenchyma (black arrow). Cr, cranial; Cd, caudal; R, right; L, left; LP, liver parenchyma

**Fig. 4:**
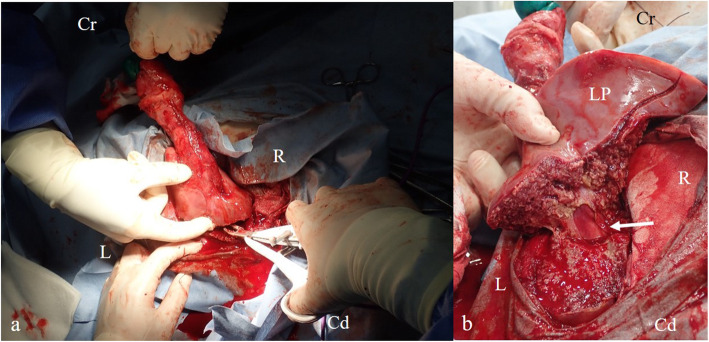
Intraoperative views of the partial hepatectomy. **a** Reset of the partial left liver robe using a vessel sealing device (LigaSure^TM^). **b** Intraoperative appearance of the portal vein (arrow). Cr, cranial; Cd, caudal; R, right; L, left; LP, liver parenchyma

For exploration of the abdominal cavity, a 15-cm right paramedian incision was performed, starting approximately 5 cm caudal to the xiphoid process and at the right side at 3 cm from the midline. At exploration of the abdominal cavity, the swollen umbilical vein, continuous from the umbilicus to the hilum of the liver, was observed. After circumcision of the umbilicus, the vein was detached from adhesions to the body wall and the greater omentum. By gentle pulling of the vein, the ventral aspects of the liver with a superficial abscess were exposed, as confirmed by intraoperative sonography (Fig. 3). Partial liver resection was initiated at the most dorsal cross section through the left liver lobe, at which no signs of abscess formation were evident sonographically. Therefore, a vessel sealing device (LigaSure^TM^ Smart Jaw; Medtronic plc, Dublin, Ireland) was used, allowing to perform repeated coagulation and cutting. The left branch of the portal vein was ligated using a polyglycolic-acid synthetic absorbable suture material (Opepolyx® USP 3, Alfresa Pharma Corp., Osaka, Japan) (Fig. 4). Visible bleeders from the cut surface of the liver were addressed by ligation with a polyglactin 910 synthetic absorbable suture material (Coated Vicryl®, USP 3-0; Ethicon, Bridgewater, NJ, USA). The resected liver weighed approximately 300 g (24% of the total liver volume) and contained two abscesses with a diameter of 2 and 4 cm, respectively. The peritoneum and muscular layer of the right midline and umbilical were closed with a continuous suture pattern using a polyglycolic-acid synthetic absorbable suture material (Opepolyx USP 3). The subcutaneous tissue was closed with a continuous suture pattern, using a polyglactin 910 synthetic absorbable suture material (Coated Vicryl, USP 0). The skin incision was ligatured with intradermal buried sutures using synthetic absorbent thread.

The pus from the resected liver abscesses was aseptically collected and subjected to aerobic and anaerobic cultures at 37°C for 24 h in a 5% sheep blood agar medium. Anaerobic culture was performed in an anaerobic jar with Anero Pack (Mitsubishi Gas Chemical Co., Inc., Tokyo, Japan). The strains isolated from the aerobic and anaerobic cultures were identified as *Corynebacterium spp.* and *Clostridium spp.* using BD BBL Crystal GP (BD Japan, Tokyo, Japan) and BD BBL Crystal ANR (BD Japan), respectively. A drug susceptibility test, which was performed as described by the Clinical and Laboratory Standards Institute, showed that *Corynebacterium spp.* was sensitive to cefazolin, while *Clostridium spp.* was sensitive to cefazolin and enrofloxacin. The calf was intravenously administered cefazolin sodium (5 mg/kg) (Cefazolin-Chu; Fujita Pharmaceutical) for 14 days to prevent postoperative infection, and flunixin meglumine (2 mg/kg) (Flunixin-Chu 10%; Fujita Pharmaceutical) for 3 days for pain relief.

**Fig. 5:**
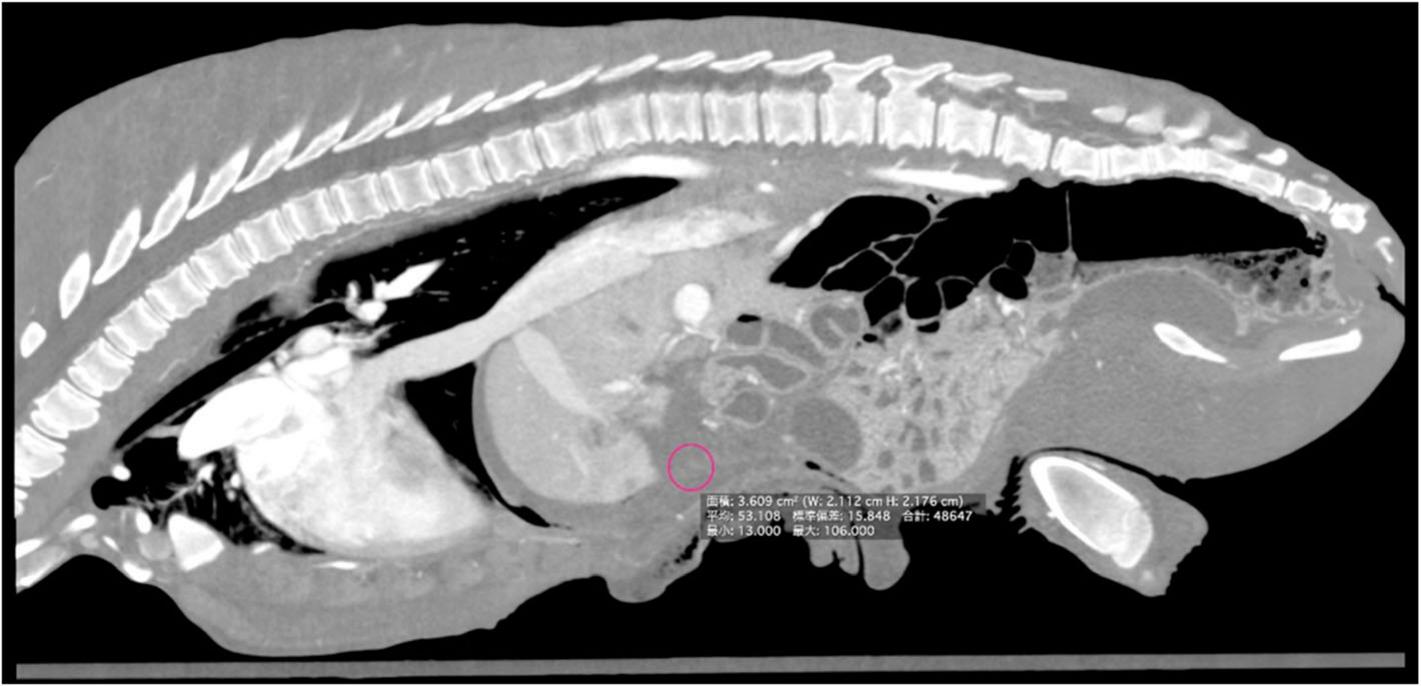
Sagittal CT image on the day following the surgery. The image depicts fluid accumulation, which appeared to be blood (CT value; 53 HU). CT, computed tomography; HU, Hounsfield units

To monitor the recovery from the partial liver resection, blood tests were performed daily from postoperative day 1 to day 5, and on postoperative day 14; abdominal ultrasonography was performed every day from postoperative day 1 to day 5, and CT scans of the abdomen were performed on postoperative days 1 and 9. On day 1 after surgery, the calf showed normal appetite, and the general condition steadily improved. The blood test results showed high levels of creatine phosphokinase and aminotransferase values until postoperative day 2 but returned to reference values on postoperative day 3. Abdominal ultrasonography revealed fluid accumulation in the ventral aspect of the abdominal cavity during the observation period. A CT scan confirmed fluid accumulation that appeared to be blood (CT value; 53 Hounsfield units) in the fundus on the day following surgery, but this was not observed on day 9 (Fig. 5). In addition, two abscesses (1.5 and 1.3 cm) that could not be removed were left in the liver. On week 3 after surgery, the general condition of the calf returned to normal. The calf returned to the farm of origin on postoperative day 38. The abscess remained at the same size at the time of discharge. Then, CT examination could not be conducted because of the relationship between the body weight and equipment, but an ultrasound examination at 6 months after surgery revealed an abscess with a diameter of approximately 1.0 cm surrounded by a thin capsule in the liver. Follow-up information was obtained after 1 year, and no complication was observed.

## Discussion and conclusions

Urachus inflammation is considered to be the most common umbilical disease, but preoperative ultrasonography revealed that the umbilical artery and urachus were retracted in this case. As the umbilical vein was swollen to 2.0 x 1.0 cm and reached the hepatic hilum, it could also result in liver abscess.

Treatment options for liver abscess include percutaneous aspiration and alcoholization, which have been reported in previous works on humans [13] and dogs [14]. These methods show no complications or recurrence and have low invasiveness and cost. Conversely, percutaneous aspiration may cause localized or systemic peritonitis [15] because of rupture of the abscess. Moreover, alcoholization may cause rupture of the abscess when the amount of ethanol injected and the injection rate are incorrect [16]. It is also difficult to be performed when the abscess is located around the portal vasculature [14]. As it has not yet been established as a safe method for cattle, it is expected that it could become a safe treatment method in the future.

The marsupialization is an option for surgical treatment in cases where the abscess has reached the hilum or in a single liver abscess in cows [5, 6]. Additionally, there is a report of partial hepatectomy in a 10-month-old Holstein heifer [12]. However, it is likely that with multiple liver abscesses, this will not adequately remove the lesions.

The bacteria remaining in the liver are considered to cause a complication; therefore, lavage of the marsupialized umbilical vein up to the liver, at an appropriate pressure, and long-term administration of an appropriate antibacterial agent, were recommended for the recovery of calves even with multiple liver abscesses [17].

In this case, the pus from the resected liver abscesses was aseptically collected and subjected to aerobic and anaerobic cultures. The strains isolated from the aerobic and anaerobic cultures were identified as *Corynebacterium spp.* and *Clostridium spp*. *Trueperella pyogenes, Escherichia coli, Proteus spp., Enterococcus, Streptococcus,* and *Staphylococcus spp* are commonly isolated in umbilical vein abscesses, which was also occurred in cases of liver abscesses [5, 18–20]. However, in cattle and in other animal species, *Corynebacterium spp.* [21, 22] and *Clostridium spp.* [23, 24] can form liver abscesses. As common bacteria are not always isolated, aerobic and anaerobic cultures should be performed in any case.

Swollen liver abscesses can damage the ventral branch of the vagus nerve and cause vagal dyspepsia [25]. Therefore, it is preferable to remove them from the body. Therefore, in our study, we aimed to remove the abscesses to the greatest extent possible and to control the remaining abscesses by long-term administration of an antibacterial drug.

Partial hepatectomy is used in humans and small animals to treat hepatocellular carcinoma [3, 26–28] and liver abscess [29–31]. During partial hepatectomy, control of bleeding and bile leakage from the cut surface is very important [32, 33]. The vessel sealing device described in this case report has previously been used for liver resection surgery in dogs [34] and in humans [33, 35], and has been found useful in reducing bleeding and bile leakage compared to the conventional clamp-crushing and open finger-fracture methods. In the current case, only minor bleeding from the hepatic transection surface was observed.

To confirm peritonitis caused by bleeding and bile leakage, which are complications of liver resection, ultrasonography of the ventral abdomen was performed for 5 days after surgery. A small amount of liquid accumulation was evident. As the blood tests did not reveal the presence of anemia, it was likely that only a very small amount of blood from the cut surface continued to be released for several days after surgery. No findings suggestive of peritonitis were obtained on CT examination on postoperative day 9.

In an experiment on healthy dogs, despite resecting 70% of the liver, the remaining tissue regenerated, and the liver returned to its original volume within approximately 2 weeks [36]. Accordingly, it is considered that even in cattle, partial hepatectomy can be performed for localized liver lesions when the liver has appropriate liver function and regeneration ability. Furthermore, even in cases of multiple liver abscesses, treatment was shown to be successful by combining surgical excision of as many abscesses as feasible and long-term administration of antibacterial agents. However, evaluation of the liver function is indicated preoperatively for proper case selection.

The painful soft tissue swelling and skin defects in the left and right carpal joints were caused by contusion due to standing aversion. CT findings showed no evidence of arthritis. Moreover, the disease was independent of the umbilical vein and liver abscess, and was cured by care with protective bandages during hospitalization. Nevertheless, infection occurred into the joint due to spread from liver abscess. The prognosis may not have been good given the associated arthritis.

To our knowledge, this was the first description of partial hepatectomy using a vessel sealing device in a calf with multiple liver abscesses. This study suggested that the combination of partial hepatectomy and long-term administration of antibacterial drugs may restore the health of calves with multiple liver abscesses.

## Data Availability

The datasets used and/or analyzed during the current study are available from the corresponding author on reasonable request.

## Abbreviation

CT: Computed tomography
